# How reproducible are the measurements of leaf fluctuating asymmetry?

**DOI:** 10.7717/peerj.1027

**Published:** 2015-06-18

**Authors:** Mikhail V. Kozlov

**Affiliations:** Section of Ecology, Department of Biology, University of Turku, Turku, Finland

**Keywords:** Environmental stress, Betula pubescens, Bioindication, Leaf fluctuating asymmetry, Research methods, Reproducibility

## Abstract

Fluctuating asymmetry (FA) represents small, non-directional deviations from perfect symmetry in morphological characters. FA is generally assumed to increase in response to stress; therefore, FA is frequently used in ecological studies as an index of environmental or genetic stress experienced by an organism. The values of FA are usually small, and therefore the reliable detection of FA requires precise measurements. The reproducibility of fluctuating asymmetry (FA) was explored by comparing the results of measurements of scanned images of 100 leaves of downy birch (*Betula pubescens*) conducted by 31 volunteer scientists experienced in studying plant FA. The median values of FA varied significantly among the participants, from 0.000 to 0.074, and the coefficients of variation in FA for individual leaves ranged from 25% to 179%. The overall reproducibility of the results among the participants was rather low (0.074). Variation in instruments and methods used by the participants had little effect on the reported FA values, but the reproducibility of the measurements increased by 30% following exclusion of data provided by seven participants who had modified the suggested protocol for leaf measurements. The scientists working with plant FA are advised to pay utmost attention to adequate and detailed description of their data acquisition protocols in their forthcoming publications, because all characteristics of instruments and methods need to be controlled to increase the quality and reproducibility of the data. Whenever possible, the images of all measured objects and the results of primary measurements should be published as electronic appendices to scientific papers.

## Introduction

Fluctuating asymmetry (FA) represents small, non-directional deviations from perfect symmetry in morphological characters. It is commonly assumed that FA results from the inability of an individual to control development while under genetic and/or environmental stress (Palmer & Strobeck, 1986; Møller & Swaddle, 1997; Leamy, 1999). The very first discoveries that FA increases in response to different disturbances led to numerous enthusiastic recommendations to use FA as a handy indicator of stress experienced by organisms (Zakharov, 1990; Clarke, 1992; Parsons, 1992; Freeman, Graham & Emlen, 1993; Hume, 2001).

However, the responses of fluctuating asymmetry (FA) to stressors are often inconsistent. In particular, more than half of the 28 studies used as examples in a recent review (Graham et al., 2010) reported a failure to detect the expected increase in bilateral FA under the impacts of natural environmental stressors. Almost every study addressing FA cites some papers that failed to show the expected effects. Furthermore, in our own analysis of a large set of samples (18 species of woody plants collected in the impact zones of 18 polluters), which were measured by research assistants who had no information on either the origin of samples or on the hypothesis being tested (i.e., blindly), showed no overall effect of industrial pollution on plant FA (Kozlov, Zvereva & Zverev, 2009).

Developing reliable and standardised methods that can be used to improve our understanding of ecological patterns and processes represents a key challenge for ecology (Bocedi et al., 2012). This is especially true for studies exploring environmental and genomic impacts on FA, because the values of FA are usually small; therefore, the reliable detection of FA requires precise measurements. However, the influences of measurement protocols on the obtained values of FA have rarely been explored. In particular, earlier studies reported differences among the observers (Yezerinac, Lougheed & Handford, 1992), including the effects of the human handedness on signed values of FA (Helm & Albrecht, 2000), and the impacts of the strategy of repeated measurements on measurement errors (David et al., 1999). Therefore, little is known regarding how much variation in the outcomes of different studies results from unavoidable differences in instruments and protocols among the scientists and research teams. The present study aimed at (i) evaluation of reproducibility of the measurements of FA in plant leaves (i.e., the consistency between the results obtained in different laboratories, by different operators and by using different instruments) and (ii) exploration of the effects of instruments and protocols used by different research teams on the obtained values of FA and on the consistency between the results of measurements performed by different researchers.

## Material and Methods

### Samples and participants

For this study, we used leaves of *Betula pubescens* Ehrh. (Betulaceae), because this tree is commonly used for studying environmental impacts on leaf FA (more than 50 papers are published to date). About 200 leaves with no traces of mechanical damage, deformation or insect feeding were collected on July 21, 2014 from a single tree of *B. pubescens* near Turku, Finland. The leaves were pressed between the sheets of filter paper and dried as ordinary herbarium specimens.

A selection of 100 perfectly preserved leaves was haphazardly divided into 10 samples, each sample (consisting of 10 leaves) was scanned with high resolution and the scale was added to each image. All 10 images were sent to 31 scientists who agreed to participate in the project (for the list of participants, consult Appendix S1). All these scientists have published studies addressing FA in plants and were therefore experienced in FA measurements.

**Figure 1:**
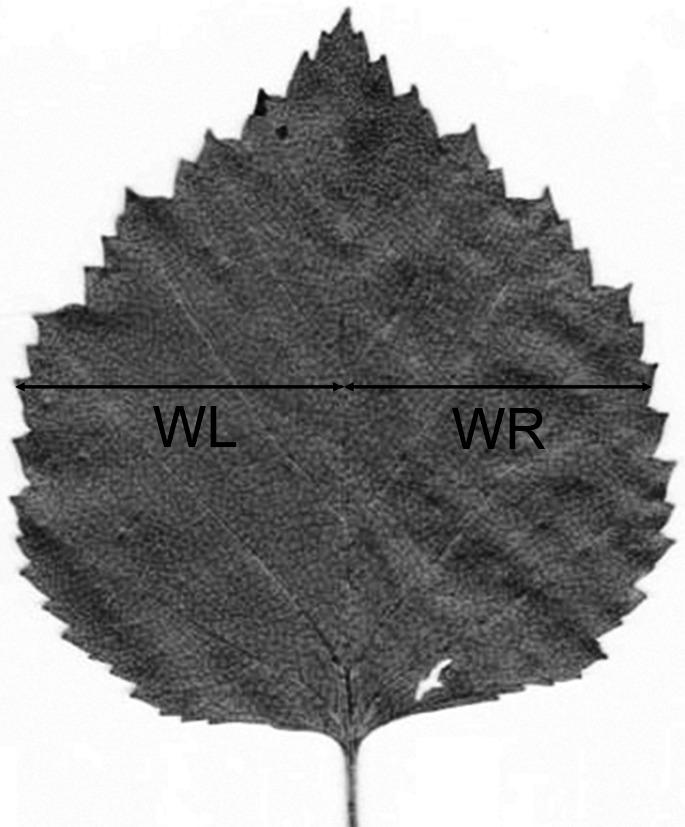
Measurements of a birch leaf for calculation of fluctuating asymmetry. Measurements of left (WL) and right (WR) halves are usually conducted perpendicular to midrib, in the middle of leaf lamina.

The participants were numbered in the order they took part in the study, and these numbers were used to identify participants across the study. The participants were asked (a) to measure the width of the left and right halves of each leaf at the midpoint between the base and the apex of leaf lamina (WL and WR; Fig. 1) using the same instruments and methods as they had used in their earlier studies and (b) to provide detailed information about the instruments and methods used in the measurements of the test samples, as well as in their earlier studies of plant FA. The character selected for this study is commonly used to quantify FA of plant leaves (more than 200 papers are published to date).

### Data analysis

Preliminary analysis demonstrated that the results by one participant greatly differed from the results of all other participants. The participant was informed about the absence of correlations between his results and the results of other participants and was asked to re-measure the test sample. The results of the first measurement were excluded from the subsequent analyses.

The values of WL and WR reported by the participants were checked for the consistency of scale. The units of measurements were converted from cm to mm whenever necessary, and all values in three of 31 data sets were multiplied by the correction coefficient to remove the systematic differences in leaf width among the participants. The mixed model ANOVA (with leaf side considered as a fixed factor and individual leaf as a random factor; SAS MIXED procedure: SAS Institute, 2009) was used to test the entire pool of data for the significance of FA relative to measurement error and for the presence of directional asymmetry (as described by Palmer & Strobeck, 1986; Palmer & Strobeck, 2003; Merilä & Björklund, 1995). The reproducibility was quantified (a) by calculating the index ME5 = [MS_*i*_ − MS_*m*_]/[MS_*i*_ + (*n* − 1)MS_*m*_], where MS_*i*_ and MS_*m*_ are the interaction and error MS from a sides × individuals ANOVA (Palmer & Strobeck, 2003), and (b) by using gauge repeatability and reproducibility (GRR) analysis (SAS VARCOMP procedure), which allows partitioning of total variance into variance of the monitored process and variance of the measurement system (Burdick, Borror & Montgomery, 2003).

The principal component analysis (PCA; SAS PRINCOMP procedure) of the signed values of FA, calculated as follows: FA = 2 × (WL − WR)/(WL + WR), was used to explore relative similarities and differences between the results obtained by the participants. The reasons for the differences among the groups of participants were identified by correlating the extracted vectors of the two first axes identified by PCA with the precision of measurements, the time spent on measurements and the numbers of leaves that were measured earlier by the researchers (SAS CORR procedure). The distribution of the absolute values of FA was greatly skewed due to a large (19.3%) numbers of zeros; therefore, the median values were compared among participants using the Kruskal-Wallis test (SAS NPAR1WAY procedure; SAS Institute, 2009).

## Results

### Instruments and protocols used to measure test samples

The data acquisition protocols applied by the participants differed in many minor but important details (Appendix S2). More than half of the participants used rulers, dividers and digital callipers to measure the width of leaf halves from the printed images of the leaves. Other participants measured leaf images using different software programs (listed in Appendix S2), including the original software that had been specifically created for the measurements of leaf FA. Only roughly half of the participants used instrumental methods to assure perpendicularity of their measurement lines with the midrib. Five participants identified the place of measurements visually, rather than by measuring leaf length and finding the exact location of the middle of the leaf lamina. Roughly half of the participants selected the starting points of their measurements away from the median line of the midrib, on the external margin of the midrib—thereby excluding the midrib width from the leaf width measurement. Most participants measured the width of the left and right halves of a leaf sequentially, but some participants obtained these measurements simultaneously, by placing the zero point of the ruler on the left leaf margin and reading two measurements—the distance to the midrib and the distance to the right leaf margin (Appendix S2). The measurement protocols applied by the participants clearly differed in many other peculiarities, which were impossible to uncover because the participants generally described their instruments and methods in insufficient detail. Some important differences in data acquisition protocols were revealed just by chance.

Seven participants slightly modified the protocol of leaf measurements. Participant 14 measured the distance from the tips of the teeth that were closest to the middle of the leaf to the point where the straight line connecting these teeth crossed the midrib. Participant 2 measured the width of the left side of a leaf in the middle of leaf lamina perpendicular to midrib, but adjusted the end point for the measurement of the right side of a leaf in such a way that this point was selected in the same position relative to marginal teeth as the end point on the left leaf margin. Participant 1 did not purposely adjust the positions of the end points relative to the teeth on leaf margins, but appreciated that this adjustment may have been done unintentionally. Five participants measured a leaf not at the midpoint between its apex and base, but at the place where the leaf width attained its highest value (Appendix S2).

Only three participants reported that the test samples were measured twice by different observers. Another 13 participants reported two measurements by the same observer, and the remaining participants conducted the measurements only once.

### Methods used for data collection in published studies

Two-thirds of the participants measured FA from fresh plant leaves or from scanned images of fresh leaves; one participant measured the images of leaves that were fixed in ethanol; and the remaining participants measured pressed and dried leaves or their images. The participants who worked with fresh leaves often bent them across the midrib (in such a way that the apex of a leaf coincided with the base) to mark the line along which the leaf halves were then measured. The participants, with nearly equal frequencies, reported long-term storage of the measured leaves, of their images and no archiving at all (or storing the leaves only until the results were published; Appendix S2).

Only three of 31 participants consistently used a blind method in their earlier studies in such a way that the persons who conducted measurements were not aware of the origin of the measured samples and/or of the hypothesis being tested.

### Identification of FA

Analysis of all measurements reported by participants (Appendix S3) demonstrated that the variation in width among the 100 measured birch leaves was highly significant (*F*_99,99_ = 45.9, *P* < 0.0001). The left and right sides of birch leaves did not differ in width (*F*_1,99_ = 0.60, *P* = 0.44), indicating an absence of directional asymmetry, whereas the highly significant side × leaf interaction (*F*_99,6000_ = 3.46, *P* < 0.0001) confirmed the existence of FA.

### Variation among the reported values of FA

**Figure 2:**
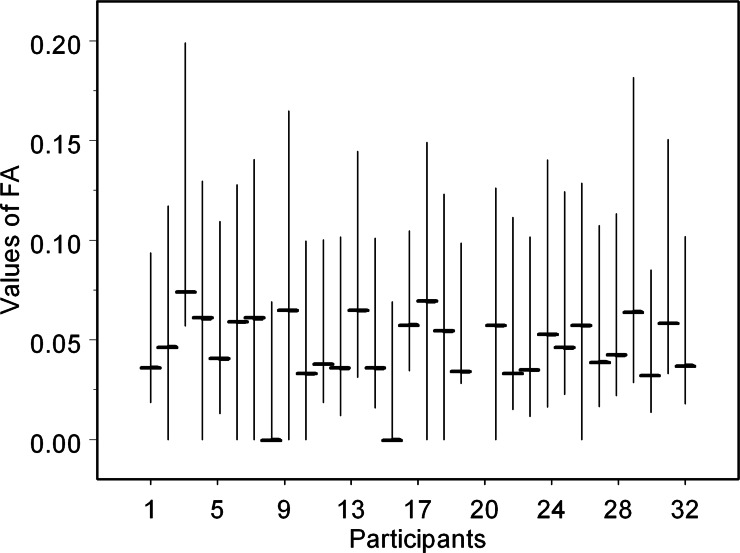
Values of fluctuating asymmetry (median, lower and upper quartiles) obtained by 31 participants from the same set of 100 birch leaves. For instruments and methods used by the participants, consult Appendix S2.

The values of FA obtained from the measurements of the same set of 100 birch leaves greatly varied among the participants (*χ*^2^ = 169.5, d.f. = 30, *P* < 0.0001): the median values ranged from 0.000 to 0.074 (Fig. 2), decreasing with the increase in the precision of measurements (*r_S_* = − 0.50, *n* = 31, *P* = 0.004).

The measurements conducted on leaf images using different software yielded the same median values of FA as obtained for the measurements conducted from the printed copies of leaf images using rulers or callipers (*χ*^2^ = 1.45, d.f. = 1, *P* = 0.22). Other recorded characteristics of the measurement protocol (Appendix S2) also had no statistically significant effect on median values of FA (*P* > 0.10).

The median values of FA did not depend on the time spent obtaining the leaf measurements (*r_S_* = 0.12, *n* = 30, *P* = 0.54) or on the experience of the participant (quantified by the number of leaves measured in the course of the earlier studies; *r_S_* = 0.05, *n* = 30, *P* = 0.77).

### Similarities and dissimilarities among the results of measurements

**Figure 3:**
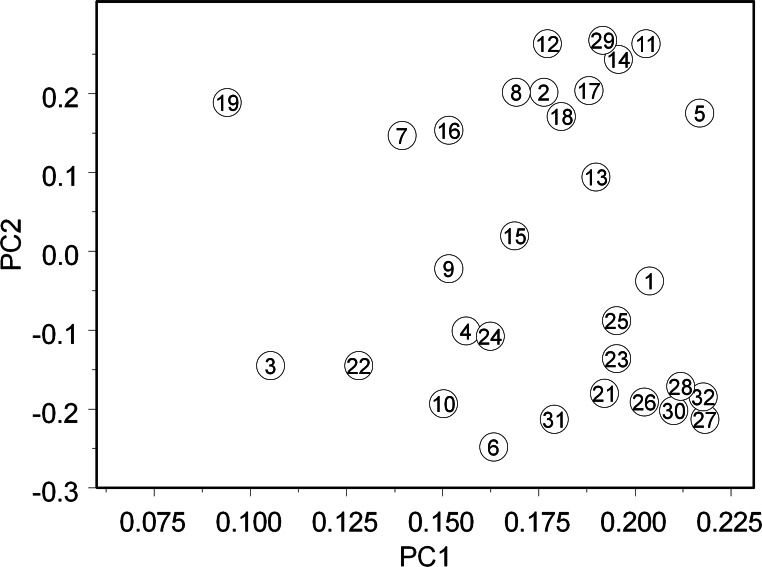
The results of principal component analysis of the signed values of fluctuating asymmetry obtained by 31 participants from the same set of 100 birch leaves. For instruments and methods used by the participants, consult Appendix S2.

The first two axes of the PCA accounted for 52.3% of the total variability among the FA values calculated from the measurements made by 31 participants (44.8% of variability along the first axis and 7.5% along the second axis; Fig. 3). The median value of the Pearson correlation coefficient between FA values based on measurements by different participants was surprisingly small (0.41), and 4.8% of all pairwise correlations were not statistically significant.

The extracted vectors of the PC1 axis were best explained by the precision of measurements (*r* = 0.44, *n* = 31, *P* = 0.02), and those of the PC2 axis by the time spent in measuring a test sample (*r* = 0.40, *n* = 30, *P* = 0.03). The visual inspection of Fig. 3 suggests that the participants can be divided into two groups that differ in their values of PC2.

### Reproducibility of the results

**Figure 4:**
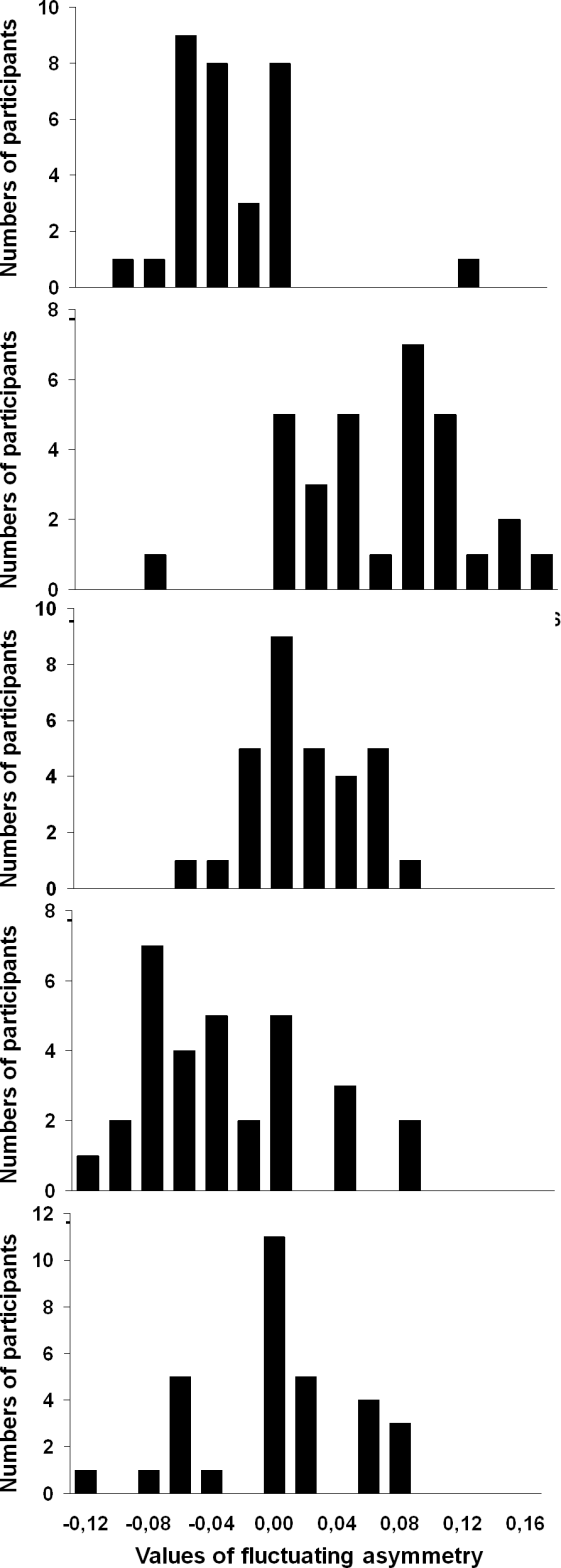
Values of fluctuating asymmetry (median, lower and upper quartiles) obtained by 31 participants from the same set of 100 birchleaves. The examples were randomly selected among 100 leaves; each panel corresponds to one leaf. Bars indicate the numbers of participants who reported FA values falling into each class interval.

The participants reported highly variable signed values of FA of individual leaves (Fig. 4). In particular, 97 of 100 leaves were reported as perfectly symmetrical (FA = 0.000) by at least one participant; the leaf-specific coefficients of variation of FA ranged from 25% to 179% (median value 84%). Nevertheless, the variation in FA among individual leaves, calculated from the results of measurements by 31 participants, was highly significant (*χ*^2^ = 595.4, d.f. = 99, *P* < 0.0001). However, the reported values of FA decreased with leaf size (*r_S_* = − 0.26, *n* = 100, *P* = 0.009); therefore, at least a part of the detected variation in FA is explained by variation in leaf size.

The overall reproducibility of the results among the participants (evaluated by the index ME5, according to Palmer & Strobeck, 2003) appeared to be very low (0.074). The GRR analysis demonstrated that the variance of the measurement system (i.e., among the participants) accounted for 79.5% of the total variance, and that the discrimination ratio—another index reflecting the quality of the measurement system—was 1.39 (CI_95_ = 1.28…1.57). Exclusion of the data provided by seven participants who modified the measurement protocol (see above) increased the reproducibility of the results (ME5 = 0.096; variance of the measurement system 59.6%; discrimination ratio 2.07).

## Discussion

Reproducibility of experimental results is a cornerstone of science (Alexander et al., 2012, and references therein). Therefore, the extremely low reproducibility of the results of the measurements of FA in a test sample of 100 leaf images, conducted independently by 31 scientists experienced in studying of plant FA, is indeed disappointing. On average, results by one participant explained only 16.8% of variation in the results of another participant. The criteria used in technology suggest that the discrimination ratio must exceed four for the measurement system to be adequate (Mader, Prins & Lampe, 1999). Furthermore, a precise measurement system (defined as one that is in statistical control with respect to variation) should be responsible for less than 10% of the total variance (BPI Consulting, 2009). The values of the discrimination ratio (1.39) and the proportion of variance due to the measurement system (79.5%) calculated from the reported measurements of a test sample indicate that the overall consistency in data acquisition protocols among the participants falls into the unacceptable category.

Interestingly, the values of repeatability/reproducibility reported in earlier publications were much higher than those found in the present study. For example, the repeatability between different assistants who measured the same sets of leaves of different plant species for our earlier study was, on average, 0.861 (Kozlov, Zvereva & Zverev, 2009). The measurements of *Robinia pseudoacacia* L. by the same researcher demonstrated repeatability from 0.374 to 0.646 for petiole lengths and from 0.990 to 0.996 for leaflet lengths (Klisarić et al., 2014). The great difference between the published estimates of repeatability and the results of the current study indicates that the second measurement performed by the same observer may eliminate occasional (technical) errors, but it is unable to detect the systematic error. The second measurement performed by another observer from the same team is more valuable: it can detect—and potentially remove—the bias associated with the personality of the observer, but not with the instruments and approaches shared between the members of a team. This was clearly demonstrated by one of the participants, who discovered and fixed a technical problem in the software used for the measurements only when s/he was informed about the absence of correlations of his results with the results obtained by the other participants.

Although two groups of participants identified by PCA (Fig. 3) differed in the time spent for measuring the test samples, the difference between the groups is likely associated with some details of the measurement protocols rather than with the time *per se*. However, the reported information (Appendix S2) did not allow identification of the proximate reasons for the differences between the groups. Nevertheless, the reproducibility increased by 30% when the data provided by the participants who modified the methods of leaf measurements were excluded from the analysis. This result suggests that further standardisation of the measurement protocol is a practicable way to achieve sufficient reproducibility of the FA values.

Standard methods based on replicate measures—which were developed to produce FA estimates as free from measurement error as possible—usually assume that the right and left sides of an individual are measured independently and are therefore subject to uncorrelated measurement errors (Palmer & Strobeck, 1986). However, this assumption is routinely discarded in measurements of leaf FA, because all participants measured both halves of a leaf before starting the measurements of the next leaf. On the other hand, David et al. (1999) concluded that the best strategy is always to take left and right values of an individual during the same session rather than in different sessions. Furthermore, many participants used the same starting point (fixed on an image) for measurements of both leaf halves, and some participants obtained the data from two leaf halves simultaneously (Appendix S2). The consequences of this variation for estimation of FA remain to be explored.

The low reproducibility of the measurements of leaf FA is in no way exceptional. The current findings on low reproducibility of biomedical results (Prinz, Schlange & Asadullah, 2011; Begley & Ellis, 2012) urged the two prestigious journals, Nature and Science, to modify their guidelines in order to improve quality control in biomedical research (McNutt, 2014; Nature editorial, 2014). Compliance with reproducibility requirements should be ensured by advising scientists working with plant FA to (i) pay utmost attention to adequate and detailed description of each point of the data acquisition protocol in their forthcoming publications, because all characteristics of instruments and methods need to be controlled to increase the reproducibility (the details of measurement protocols summarised in Appendix S2 may be used as a template); (ii) use blind methods—most importantly, the person conducting measurements should not be aware of the origin of samples being measured and of the hypothesis being tested (Kozlov & Zvereva, 2015); (iii) archive the images of all measured objects and the results of primary measurements and, whenever possible, publish these materials as electronic appendices to scientific papers; and (iv) measure the widths of leaf halves at least to the nearest 0.5 mm, because measurements conducted to the nearest 1 mm tend to overestimate FA and decrease reproducibility. Implementation of these recommendations will provide readers with a clear understanding of how the data were collected. This, in turn, would increase the credibility of the published results. More generally, the researchers studying FA should agree upon a kind of standards for various measurements, and the present study can be seen as the first step towards this goal.

What is the next step? Drawing conclusions about the low reproducibility of studies exploring FA in plants, based on the unacceptably low reproducibility of primary measurements, is risky. One might argue that when a systematic error similarly affects the values of FA in the control group and in the treatment group, the difference between these groups can still be revealed reliably. The group of scientists who participated in the measurements of test samples (see Appendix S1) agreed to continue their co-operation in order to check the reproducibility of the published studies on plant FA. We would warmly welcome all researchers who would like to join in this project, which should potentially provide recommendations on data acquisition protocols aimed at minimising variations among scientists and thus increasing reproducibility of studies addressing plant FA.

## Supplemental Information

10.7717/peerj.1027/supp-1 Appendix S1 The list of scientists who contributed to the study by conducting measurements of test samples

10.7717/peerj.1027/supp-2 Appendix S2 Characteristics of methods used in measurements of fluctuating asymmetry by project participants

10.7717/peerj.1027/supp-3 Appendix S3 Results of measurements reported by project participants Data format: participant number (matches Appendix S2); sample number; leaf number; width of the leaf half; width of the right half.
