# Supplementary material for: How reproducible are the measurements of leaf fluctuating asymmetry?
Source: PeerJ. 2015 Jun 18;3:e1027. doi: 10.7717/peerj.1027 (PMC4476141; doi:10.7717/peerj.1027)
Supplement: Appendix S1 [file peerj-03-1027-s001.doc]

**Appendix A.** The list of scientists who contributed to the study by conducting measurements of test samples

Ambo-Rappe R. – Department of Marine Science, Faculty of Marine Science and Fisheries, Hasanuddin University, Tamalanrea Km. 10, Makassar 90245, Indonesia

Amosova I. B. – Department of Botany, Basic Ecology and Nature Management, Northern (Arctic) Federal University, Severnaya Dvina Emb. 17, 163000 Arkhangelsk, Russia

Anonymous[[1]](#footnote-2) – Krasnoyarsk, Russia

Belyaeva Yu. V. – Department of Modern Life Sciences, Volga Region State University of Service, Gagarina 4, 445677 Tolyatti, Russia

Cornelissen T. – Departmento of Natural Sciences, Universidade Federal de Sao Joao Del-Rei, Sao Joao Del-Rei-MG, Brazil 36301-160

Cuevas-Reyes P. – Laboratorio de Ecología de Interacciones Bióticas, Facultad de Biología, Universidad Michoacana de San Nicolás de Hidalgo, Ciudad Universitaria, 58030, Morelia, Michoacán, Mexico

Costa, F.V. – Universidade Federal de Minas Gerais, Instituto de Ciências Biológicas, Av. Antônio Carlos 6627, Laboratório de Ecologia de Insetos/Laboratório de Ecologia de Mutualismos, Pampulha, 31270901 Belo Horizonte, MG, Brasil

Ereschenko O. V. – Department of Ecology, Biochemistry and Biotechnology, Altai State University, Lenina 61, Barnaul, 656049 Russia

Gavrikov D. E. – Department of Natural Sciences, Pedagogical institute, Irkutsk State University, Nizhnyaya Naberezhnaya 6, 664011 Irkutsk, Russia

Glinyanova I. Yu. – Department of Safety of Life Activities in Technosphere, Volgograd State University of Architecture and Civil Engineering, Akademicheskaya 1, 400074 Volgograd, Russia

Ibragimova E.E. – Crimean Engineering-Pedagogical University, Uchebnyi Pereulok 8, 295015 Simferopol, Russia

Kaligaric M. – Department of Biology, Faculty of Natural Sciences and Mathematics, University of Maribor, Koroška cesta 160, 2000 Maribor, Slovenija

Komac B. – CENMA - Institut d'Estudis Andorrans, Avinguda Rocafort 21-23, AD600 Sant Julià de Lòria - Principat d'Andorra

Korotchenko I. S. – Department of Ecology and Natural Sciences, Krasnoyarsk State Agrarian University, Mira Avenue 90, 660049 Krasnoyarsk, Russia

Lozinskaya O. V. – Department of Ecological and Molecular Genetics, International Sakharov Environmental University, **Dolgobrodskaya Street 23, 220070 Minsk, Belarus**

Marchenko S. I. – Department of Forest Cultures and Soil Science, Bryansk State Technological Academy of Engineering, Stanke Dimitrova 3, 241037 Bryansk, Russia

Milligan J. R. – Department of Biological, Geological and Environmental Sciences, Cleveland State University, 2121 Euclid Avenue, Cleveland, OH 44115-2214, U.S.A.

Nuche Gálvez P. – Pyrenean Institute of Ecology (CSIC), Avda. Montañana 1005, P. O. Box 13034, 50059 Zaragoza, Spain

Smirnova N.A. – Department of Geoecology and Nature Management, St. Petersburg State University, Universitetskaya Naberezhnaya 7-9, 199034 St. Petersburg, Russia (supervised by Opekunova M. G. from the same university)

Pospelova O. A. – Stavropol State Agrarian University Zootechnicheskaya 12, 355017 Stavropol, Russia

Rodikova A. V. – Tomsk State University, Lenina 36, 634050 Tomsk, Russia

Rossi M. N. – Laboratório de Ecologia Populacional, Universidade Federal de São Paulo (Unifesp), Departamento de Ciências Biológicas, Rua Arthur Riedel, 275, Eldorado 09972-270, Diadema, SP, Brasil

Santos J. C. – Laboratório de Ecologia e Desenvolvimento de Galhas, Instituto de Biologia (InBio) - Universidade Federal de Uberlândia (UFU), Av. Pará 1720 - Cep 38400-902. Uberlândia, Minas Gerais, Brasil

Sarsatskaya A. S. – Department of Human Physiology and Safety of Life Activities, Kemerovo State University, Krasnaya 6, 650043 Kemerovo, Russia

Shadmanova T. Kh. – Ecostandard Ltd., Chernyshevskogo 14, 414000 Astrakhan, Russia (supervised by Chuikov Yu. S. – Department of Ecology and Safety of Life Activities, Astrakhan State University, Tatistcheva 20a, 414056 Astrakhan, Russia).

Simonova Z. A. – Department of Ecology, Yuri Gagarin State Technical University of Saratov, Politechnicheskaya street 77, 410054 Saratov, Russia

Skochilova E. A. – Departmant of Ecology, Mari State University, Lenin Square 1, 424000 Yoshkar-Ola, Russia

Soldatova V.Yu. – Institute of Natural Sciences, North-Eastern Federal University, Belinskogo 58, 677890 Yakutsk, Russia (supervised by Shadrina E.G. from the same institute)

Timokhina O. A. – Nizhnetagilskaya State Social and Pedagogical Academy, Krasnogvardeyskaya 57, 622031 Nizhnyi Tagil, Russia

Wuytack T. – Department of Bioengineering, University of Antwerp, Campus Groenenborger, Groenenborgerlaan 171, G.V.623, 2020 Antwerpen, België

Yousefzadeh H. – Department of Forestry, Faculty of Natural Resources, Tarbiat Modares University (TMU), Noor 46417-76489, Mazandaran Province, Iran

1. The participant, after reading the manuscript, requested the removal of personal information from this list. [↑](#footnote-ref-2)
