# Supplementary material for: How reproducible are the measurements of leaf fluctuating asymmetry?
Source: PeerJ. 2015 Jun 18;3:e1027. doi: 10.7717/peerj.1027 (PMC4476141; doi:10.7717/peerj.1027)
Supplement: Appendix S2 [file peerj-03-1027-s002.doc]

**Appendix S2.** Characteristics of methods used in measurements of fluctuating asymmetry of plant leaves

Part 1. Instruments and methods used in measurements of test samples

| No^a^ | The measured object | Instruments used^b^ | Preci-sion, mm^d^ | Finding the middle of the midrib^e^ | Mark on the middle of the midrib | Perpendi-cularity to the midrib^f^ | Measurements of two halves of a leaf: | | | | Time spent^l^, hours | Number of leaves measured earlier^m^ |
| --- | --- | --- | --- | --- | --- | --- | --- | --- | --- | --- | --- | --- |
|  |  |  |  |  |  |  | Along the straight line^g^ | Starting point(s)^h^ | From the same point^i^ | Indepen-dence of measure-ments^k^ |  |  |
| 1 | Image | Adobe Photoshop CS3 (software) | 0.025 | Exact | Yes | Approx. | No | C | No | Yes | 6.0 | 3000 |
| 2 | BW print | Ruler | 0.5 | Exact | Yes | Exact | Yes | M | No | Yes | 1.5 | 750 |
| 3 | BW print | Ruler | 1.0 | N/A | Yes | Exact | Yes | C | No | Yes | 1.4 | 5000 |
| 4 | BW print | Ruler & set square | 1.0 | Exact | Yes | Exact | Yes | M | No | Yes | 0.5 | 25000 |
| 5 | Image | Surfer 8 (software) | 0.01 | Exact | No | Exact | Yes | С | Yes | No | 4.0 | 7500 |
| 6 | BW print | Divider & ruler | 1.0 | Exact | Yes | Approx. | Yes | C | No | Yes | 1.5 | 500 |
| 7 | BW print | Ruler | 1.0 | Exact | Yes | Exact | Yes | M | No | Yes | 2.5 | 5000 |
| 8 | BW print | Divider & ruler | 0.5 | Exact | Yes | Exact | Yes | C | Yes | No | 1.8 | 200 |
| 9 | BW print | Ruler & set square | 1.0 | Exact | Yes | Exact | Yes | M | No | Yes | 2.0 | 5000 |
| 10 | Colour print | Ruler | 0.5 | Exact | Yes | Exact | Yes | C | Yes | No | 1.0 | 25000 |
| 11 | Image | PhotoM 1.21 (software) | 0.1 | Exact | No | Approx. | No | C | Yes | No | 2.0 | 500 |
| 12 | Colour print | Digital caliper (0.1 mm scale) | 0.1 | Exact | Yes | Approx. | Yes | C | Yes | No | 1.5 | 10000 |
| 13 | Colour print | Ruler | 1.0 | Exact | Yes | Approx. | No | С | No | Yes | 2.5 | 10000 |
| 14 | Image | Bio (software)^c^ | 0.001 | Exact | No | Approx. | No | C | Yes | No | 3.0 | 50000 |
| 15 | Colour print | Ruler | 0.5 | Exact | Yes | Exact | Yes | M | No | Yes | 1.2 | 10000 |
| 16 | Image | Pendula (software)^c^ | 0.01 | Exact | No | Exact | Yes | C | Y | No | 3.5 | 100000 |
| 17 | BW print | Ruler & set square | 1.0 | Exact | Yes | Exact | Yes | C | Yes | No | 1.7 | 3000 |
| 18 | Image | Ruler | 1.0 | Exact | No | Approx. | No | C | No | Yes | 1.5 | 5000 |
| 19 | Colour print | Ruler | 0.5 | Exact | Yes | Exact | Yes | M | No | Yes | 6.0 | 1000 |
| 21 | Image | Ruler | 1.0 | N/A | No | Approx. | No | C | No | Yes | 2.0 | 0 |
| 22 | Image | UTHSCA Image Tool (software) | 0.01 | N/A | Yes | Approx. | Yes | C | Yes | No | 1.2 | 15000 |
| 23 | Image | Image J (software) | 0.01 | N/A | Yes | Exact | Yes | C | Yes | No | 2.8 | 360 |
| 24 | Image | Image J (software) | 0.01 |  | . | . | . | . | . | . | 2.0 | 300 |
| 25 | BW print | Digital caliper (0.01 mm scale) | 0.01 | Approx. | Yes | Approx. | Yes | C | Yes | No | 0.5 | 1000 |
| 26 | Colour print | Ruler | 1.0 | Exact | Yes | Exact | Yes | M | No | Yes | 2.0 | 100 |
| 27 | Image | SigmaScan Pro 5 (software) | 0.01 | Approx. | Yes | Approx. | Yes | C | Yes | No | 0.9 | 690 |
| 28 | Image | ImageTool 3.00 (software) | 0.01 | N/A | No | Approx. | Yes | C | Yes | No | 5.0 | 750 |
| 29 | Image | Image Pro Plus 5.0 (software) | 0.01 | Exact | Yes | Exact | Yes | M | No | Yes | 5.0 | 540 |
| 30 | Image | Leica image Analysis (software) | 0.01 | Approx. | Yes | Approx. | No | M | No | Yes | 1.0 | 600 |
| 31 | Image | SigmaScan Pro (software) | 0.1 | Approx. | Yes | Approx. | Yes | M | Yes | No | 2.0 | 10500 |
| 32 | Image | ImageJ (software) | 0.01 | Approx. | No | Approx. | No | M | No | Yes | 0.7 | 700 |

^a^ Participants were numbered in the order they took part, and these numbers were used to identify participants across the study. Participant 20 did not provide the results of the measurements, and participant 24 did not provide information on instruments and methods.

^b^ The rulers used by participants always had 1 mm scale.

^c^ Software was specifically developed for measurements of leaf fluctuating asymmetry – for internal use only.

^d^ Concluded from the reported measurements.

^e^ Exact – by measurements or by folding a leaf across the midrib (in such a way that the apex of a leaf coincides with the base); approx. – visually; N/A – not applicable: the participant measured the width of leaf halved in the widest place of leaf lamina..

^f^ Exact – by folding a leaf across the midrib, by set square or by software instruments; approx. - visually.

^g^ Yes – the angle between the lines, along which the measurements of leaf halves were made, is always 180˚, even if this line is not perpendicular to the midrib; no – the angle between the lines, along which the measurements of leaf halves were made, may differ from 180˚.

^h^ C – at the middle of the midrib, M – at the external margin of the midrib.

^I^ Yes – the measurements from two halves of a leaf always started from exactly the same point; no – the starting points from the measurements of the left and right halves of a leaf were selected independently from each other.

^k^ Yes – the measurements of two halves of a leaf were independent from each other; No – the measurements were to a certain extent dependent from each other (they started from the same point or were recorded simultaneously, without changing the position of the measuring device).

^l^ Time spent for the measurements of test samples (100 birch leaves).

^m^ Approximate; used as an index of the experience of the participant.

Part 2. Instruments and methods used in earlier studies

| No^a^ | Leaves used | Folding/cutting of leaves | Measured objects | Sample archiving | Measurements conducted by^c^ | Knowledge^d^ on | | Number of measu-rements/measurers |
| --- | --- | --- | --- | --- | --- | --- | --- | --- |
|  |  |  |  |  |  | Leaf origin | Hypothesis |  |
| 1 | Pressed and dried | No | Colour images | No | Permanent assistants | Yes | Sometimes | 2, same person |
| 2 | Pressed and dried | No | Leaves | No | Author | Yes | Yes | 1 |
| 3 | Fresh | No | Leaves | Leaves^b^ | Author | Yes | Yes | 2-3, same person |
| 4 | Fresh | Bent across midrib | Leaves | No | Author | Yes | Yes | 1 |
| 5 | Fresh | Cut across midrib | Colour images | Leaves (part.) | Author, permanent assistants | Yes | Sometimes | 1 |
| 6 | Fresh | No | Leaves | No | Temporary assistants | Yes | No | 2, same person |
| 7 | Fresh | Bent across midrib | Leaves | No | Author, temporary assistants | Sometimes | Sometimes | 1 |
| 8 | Fresh | Bent across midrib | Leaves | No | Author, temporary assistants | Yes | Sometimes | 1 |
| 9 | Fresh | Bent across midrib | Leaves | No | Author | Yes | Yes | 1 |
| 10 | Fresh | Bent across midrib | Leaves | No | Author | Yes | Yes | 1 |
| 11 | Fresh | Bent along midrib | Leaves | Leaves | Author, temporary assistants | Yes | Yes | 2, different persons |
| 12 | Fresh | No | Leaves | Leaves^b^ | Author | Yes | Yes | 1 |
| 13 | Fresh | Bent across midrib | Leaves | Leaves | Author | Yes | Yes | 2, same person |
| 14 | Fixed in ethanol | No | Colour images | Leaves^b^, images | Permanent assistants | No | No | 1 |
| 15 | Fresh | Bent across midrib | Leaves | No | Author, permanent assistants | Sometimes | Yes | 2, same or different person |
| 16 | Fresh | No | Colour images | Images | Author, permanent assistants | Yes | Yes | 2, same person |
| 17 | Fresh | Bent across midrib | Leaves | Leaves^b^ | Author | Yes | Yes | 2, same person |
| 18 | Pressed and dried | No | Leaves or colour images | Images | Author, temporary assistants | Yes | Yes | 1-2, different persons |
| 19 | Fresh | Bent across midrib | Leaves | Leaves^b^ | Author, temporary assistants | Yes | Yes | 2+, different persons |
| 21 | Pressed and dried | No | Colour images | Images | Temporary assistants | Yes | Yes | 2, same person |
| 22 | Pressed and dried | No | Colour images | Images | Author, temporary assistants | Sometimes | Yes | 1 |
| 23 | Pressed and dried | No | Leaves or colour images | Leaves | Author | Yes | Yes | 2, same person |
| 24 | . | . | . | . | . | . | . | . |
| 25 | Pressed and dried | No | Colour images | Images | Author, temporary assistants | Yes | Yes | 1 |
| 26 | Pressed and dried | No | Leaves | Leaves^b^ | Author, temporary assistants | Sometimes | Yes | 2, same person |
| 27 | Fresh | Glued to paper | Colour images | Images | Author, temporary assistants | No | Yes | 1 |
| 28 | Fresh | No | Colour images | Images | Author | Yes | Yes | 2, same person |
| 29 | Pressed and dried | No | Colour images | Leaves | Author | Yes | Yes | 2, same person |
| 30 | Fresh | No | Colour images | Images | Temporary assistants | Yes | Yes | 1-2, same person |
| 31 | Pressed and dried | No or bent along midrib | Colour images | Images | Temporary assistants | No | No | 1 |
| 32 | Fresh | Cut along midrib | Colour images | Images | Author | Yes | Yes | 2, same person |

^a^ Participants were numbered in the order they took part, and these numbers were used to identify participants across the study. Participant 20 did not provide the results of the measurements, and participant 24 did not provide information on instruments and methods.

^b^ Short-term archiving (until publication of the results).

^c^ Permanent assistants are defined as specifically trained staff members; temporary assistants are usually students instructed how to conduct the measurements.

^d^ Yes – the person conducting the measurements was aware of the origin of leaves and/or of the hypothesis being tested, i.e., the method was not blind; no – the person conducting the measurements was not informed on the origin of leaves and/or of the hypothesis being tested, i.e., the method was blind.
